# Diverging Concepts and Novel Perspectives in Regenerative Medicine

**DOI:** 10.3390/ijms18051021

**Published:** 2017-05-09

**Authors:** Maurizio Muraca, Martina Piccoli, Chiara Franzin, Anna Maria Tolomeo, Marcin Jurga, Michela Pozzobon, Giorgio Perilongo

**Affiliations:** 1 Department of Women’s and Children’s Health, University of Padova, Via Giustiniani 3, 35128 Padova, Italy; annamaria.tolomeo@hotmail.it (A.M.T.); m.pozzobon@irpcds.org (M.P.); giorgio.perilongo@unipd.it (G.P.); 2 Institute of Pediatric Research “Città della Speranza”, Corso Stati Uniti 4, 35127 Padova, Italy; m.piccoli@irpcds.org (M.P.); c.franzin@irpcds.org (C.F.); 3 The Cell Factory, a Company of Esperite, Galileilaan 19, 2845 Niel, Belgium; marcin.jurga@cell-factory.com

**Keywords:** stem cells, pluripotent stem cells, autologous cell transplantation, allogenic cell transplantation, cell therapy, paracrine signaling, extracellular vesicles, industry manufacturing

## Abstract

Regenerative medicine has rapidly evolved, due to progress in cell and molecular biology allowing the isolation, characterization, expansion, and engineering of cells as therapeutic tools. Despite past limited success in the clinical translation of several promising preclinical results, this novel field is now entering a phase of renewed confidence and productivity, marked by the commercialization of the first cell therapy products. Ongoing issues in the field include the use of pluripotent vs. somatic and of allogenic vs. autologous stem cells. Moreover, the recognition that several of the observed beneficial effects of cell therapy are not due to integration of the transplanted cells, but rather to paracrine signals released by the exogenous cells, is generating new therapeutic perspectives in the field. Somatic stem cells are outperforming embryonic and induced pluripotent stem cells in clinical applications, mainly because of their more favorable safety profile. Presently, both autologous and allogeneic somatic stem cells seem to be equally safe and effective under several different conditions. Recognition that a number of therapeutic effects of transplanted cells are mediated by paracrine signals, and that such signals can be found in extracellular vesicles isolated from culture media, opens novel therapeutic perspectives in the field of regenerative medicine.

## 1. Introduction

Regenerative medicine is a young discipline that has rapidly evolved during the last twenty years due to the remarkable progress of cell and molecular biology, which has paved the way to the use of living cells (particularly stem cells) as therapeutic tools for a variety of diseases. Expectations from both patients and physicians have led to an accelerated transfer of these novel therapies from bench to bedside without sufficient knowledge of the pathophysiological mechanisms involved. Thus, inconsistent outcomes of the first phase II–III clinical trials of cell therapy have resulted in severe financial losses and reduced confidence in the possibilities of the field. According to the “Gartner’s curve”, ground-breaking innovations may result in a peak of inflated expectations, followed by a trough of disillusionment when they fail to deliver as promised. However, if the innovation is based on solid foundations, it is possible to learn from previous mistakes and open a new positive phase, the “plateau of productivity” [1]. Regenerative medicine seems to be now in this phase, marked by the commercialization of the first cell therapy products following approval by regulatory authorities and by the entry of big pharma into the field. Possibly, improved patient selection and better knowledge of tissue biology (i.e., host tissue interaction with transplanted cells) has helped entrance into this new phase. However, several unresolved issues are still a matter of debate, including the preferential use of pluripotent vs. somatic, and of allogenic vs. autologous stem cells. In addition, the prevailing concept of stem cells as “spare parts” has now been modified by the acknowledgment that, in several instances, the observed beneficial effects of cell transplantation are not due to integration and differentiation of transplanted cells into the host tissue, but rather result from complex paracrine signals released by the exogenous cells themselves.

## 2. Pluripotent vs. Somatic Stem Cells

Pluripotent stem cells (PSCs) represent the original revolutionary promise of regenerative medicine, because of their ability to self-renew indefinitely, and because in theory they can also differentiate into any cell type in the body, thus providing functional replacement or trophic support to dysfunctional cells and tissues in various diseases. Expectations for their therapeutic application in regenerative medicine date back almost two decades, when human embryonic stem cells (hESCs) were first derived from the inner cell mass of embryos [2]. Researchers throughout the world spent considerable efforts improving expansion and differentiation protocols, in order to obtain large amounts of the desired cell phenotype free of pluripotent leftovers, thus minimizing the risk of teratoma formation. Following extensive research in animal models, early clinical applications were concentrated in the areas of retinal diseases, cardiac ischemia, diabetes mellitus, and spinal cord injuries [3]. Retinal diseases are a preferential area of application since the eye is believed to be an immune privileged site, and being an isolated organ, it allows local administration of the cells with minimal systemic distribution. In a recent report, a significant vision improvement was demonstrated in the majority of 18 patients with Age-related Macular Degeneration (AMD) or with Stargardt’s Macular Dystrophy, a disease resulting in retinal cell degeneration and central vision loss in children after the age of ten [4]. Transplanted patients were followed up for a median of 22 months by serial systemic, ophthalmic, and imaging examinations. There was no evidence of adverse proliferation, rejection, or serious ocular or systemic reactions, and adverse events were associated with vitreoretinal surgery and immunosuppression. Thirteen out of 18 patients (72%) had patches of increasing subretinal pigmentation, consistent with transplanted retinal pigment epithelium. Best-corrected visual acuity, monitored as part of the safety protocol, improved in ten eyes, improved or remained the same in seven eyes, and decreased by more than ten letters in one eye, whereas the untreated fellow eyes did not show similar improvements in visual acuity. Vision-related quality-of-life measures increased after transplantation for general and peripheral vision, as well as near and distance activities, both in patients with AMD and in patients with Stargardt’s macular dystrophy.

The technology of induced pluripotent stem cells (iPSCs) developed by Yamanaka [5] involves the reprogramming of differentiated somatic cells into a pluripotent state by the introduction of a cocktail of factors to “reset” the transcriptional program of the cell back to an embryonic state. The aims were the generation of autologous, and thus non-immunogenic, patient-specific cell lines for transplantation, as well as to avoid ethical concerns related to the use of human embryos. Following encouraging results in non-human primates, the first in-man clinical iPSC-based trial was announced in 2014, aimed at treating patients affected by the exudative form of AMD with autologous iPSC-derived retinal pigment epithelium (RPE) sheets [3]. In the first treated patient, the RPE sheet transplanted under the macula survived well without any findings of immune rejection nor of excessive proliferation, and no significant adverse event was observed during a one-year follow-up. Retinal imaging showed improvement of the exudative alterations, best corrected visual acuity was maintained without additional anti-VEGF therapy, and the score of the Visual Function Questionnaire improved. A second patient was then enrolled, but her iPSCs reportedly contained a mutation, potentially in an oncogene. Thus, the trial was put on hold pending a possible redesign [6].

A major obstacle in the development of iPSC-based therapies is the level of artificial manipulation. ESCs first need to be artificially maintained and propagated in an undifferentiated status, and are then directed towards a specific differentiation pathway. For the generation of iPSCs, somatic cells are reprogrammed to a pluripotent state, and then differentiated as above. The whole process thus generates a series of risk factors (for a review, see e.g., [7,8]).The risk of teratoma formation is related to the contamination of human iPSC-derived differentiated cells with residual undifferentiated and pluripotent cells. Chromosomal aberrations can already be present in the somatic cells before reprogramming (especially when derived from aged individuals), or may result from the reprogramming process, or from culture conditions [9]. Indeed, it has been reported that the karyotype of human iPSCs can become unstable during prolonged culture in vitro, exhibiting resistance to apoptosis, altered differentiation patterns, and persistent stem cells populations in teratomas [10]. Moreover, several oncogenes were found to be overexpressed in iPSCs [11,12]. Further research led to the development of alternative, virus-free derivatization methods, limiting the tumorigenic risk associated with the genomic integration of reprogramming factors. These non-integrating reprogramming protocols, in particular mRNA-based techniques, guaranteed higher efficiency, improved reliability in terms of success rate and low frequency of possible de novo genetic aberrations, giving rise to iPSCs displaying epigenetic patterns close to those of ESCs lines and able to differentiate into the three germ layers [13]. Recently, Luni and colleagues described a method that combined the use of non-integrating modified mRNAs with a microfluidic chip, enabling automated and highly efficient reprogramming of human somatic cells to iPSCs, to date with the highest efficiency reported in literature. These so-called µ-hiPSC are highly homogeneous, with 85% of cells expressing pluripotency markers, are karyotypically normal after expansion for 12 passages, and can also be differentiated on-chip into different cell types [14]. Improved clinical safety could possibly be reached by purifying iPSCs-derived differentiated cells using fluorescence-activated cell sorting (FACS) with antibodies against pluripotency markers [15] or by other techniques such as chemical ablation by targeting tumorigenicity-associated proteins or by discriminating undifferentiated iPSCs for their different electrochemical potential [16]. Moreover, next-generation sequencing techniques could be applied in order to establish routine tests for the genetic integrity of iPSCs produced for therapeutic application [7].

Still, these cell products are subjected to a high level of scrutiny. As stated above, clinical trials involving local cell administration in a contained environment are preferred due to limited availability of data on toxicity and biodistribution, as well as because of concerns regarding contamination with residual pluripotent cells, tumorigenic risks, and ectopic tissue formation.

Somatic stem cells (SSCs) are considered safer therapeutic tools, since in theory they can be administered without further manipulation following isolation from tissues. In practice, with the exception of hematopoietic stem cells (HSCs), in vitro expansion is required in order to obtain sufficient amounts for therapeutic applications, a process that might result in genetic abnormalities. Not unexpectedly, in vitro expansion yields reproducible results with high turnover tissues such as epithelia, paving the way for the successful cell therapy of cutaneous and corneal lesions [17,18]. However, mesenchymal stem/stromal cells (MSCs) now seem to represent the most promising tool in Regenerative Medicine, even surpassing HSCs in the scientific literature (4435 publications on MSCs vs. 3797 publications on HSCs in the year 2015). Current experimental therapeutic applications mainly exploit the immune modulatory properties of MSCs [19], as well as their ability to protect organs and tissues from a variety of injuries favoring regeneration with restitutio ad integrum [20]. Currently, 271 open, recruiting interventional studies using MSCs (conditions: NOT tumor, cancer, proliferative disorders) are registered at the National Institute of Health website (clinicaltrials.gov). Target immune disorders include graft-versus-host disease (GVHD), diabetes, inflammatory bowel disease, multiple sclerosis, amyotrophic lateral sclerosis, Sjögren syndrome, rheumatoid arthritis, systemic sclerosis and systemic lupus erythematosus. These figures clearly indicate an enduring confidence in the therapeutic possibilities offered by MSCs, even in the face of limited clinical evidence. Importantly, a recent meta-analysis supported the safety of MSC-based treatments after evaluating 36 studies, eight of which were randomized controlled trials [21]. Target diseases included ischemic stroke, Crohn’s disease, cardiomyopathy, myocardial infarction, GVHD and healthy volunteers, with a total of 1012 participants. No association was found between MSCs and acute infusion-related toxicity, organ system complications, infection, death or malignancy. The only significant association was between MSC infusion and transient fever. However, larger scale controlled clinical trials with rigorous reporting of adverse events are required to further define the safety profile of MSCs. Most clinical studies are uncontrolled phase I/II trials enrolling a limited number of patients, sometimes under poorly defined conditions, and reports of failures have been frequent [22,23]. Some positive results are, however, being produced in larger phase III or in better conducted phase II studies.

A phase III double-blind, placebo-controlled clinical trial using commercial allogeneic MSCs (Prochymal^®^, Osiris Therapeutics Inc., Columbia, MD, USA) for the treatment of GVHD included two different protocols [24]. The first protocol evaluated the safety and efficacy of Prochymal in association with steroid therapy in 192 patients. No significant beneficial effect on 90-day survival was observed in this patient population. The second protocol evaluated the safety and efficacy of Prochymal in patients resistant to corticosteroid therapy. The primary endpoint was a sustained complete response for at least 28 days. This endpoint was only met in the subgroups of patients with steroid-refractory hepatic and gastrointestinal GVHD. Importantly, Prochymal showed a stronger trend of improvement in response rates in pediatric patients (86% vs. 57%, *p* = 0.094, *n* = 28). No adverse events were registered. Despite the mixed results of the trial, on May 2012, the sponsor company received market authorization from Canada Health Authorities for the treatment of steroid-resistant GVHD in pediatric patients, making Prochymal the world’s first approved drug having stem cells as its active ingredient. Additional evidence of efficacy of the drug in this pediatric population was provided in further studies [25].

A multicenter randomized, double-blind trial recruited 212 Crohn’s disease patients with complex perianal fistulas with inadequate response to previous therapies, including anti-tumor necrosis factors (TNFs). Patients were randomized to receive either placebos, or a single intralesional injection of 120 million allogeneic, expanded, adipose-derived stem cells (Cx601) [26]. The primary endpoint of the study was combined remission at 24 weeks, and it was analyzed again at 52 weeks as a secondary variable. A significantly greater proportion of patients treated with Cx601 versus placebo, achieved combined remission in the intention-to-treat (ITT) population (53 of 107 (50%) vs. 36 of 105 (34%); difference 15.2%, 97.5% CI 0.2–30.3; *p* = 0.024) and modified ITT populations (53 of 103 (51%) vs. 36 of 101 (36%); 15.8%, 0.5–31.2; *p* = 0.021). 18 (17%) of 103 patients in the Cx601 group versus 30 (29%) of 103 in the placebo group experienced treatment-related adverse events, the most common of which were anal abscess (six in the Cx601 group vs. nine in the placebo group) and proctalgia (five vs. nine). Of note, 75% of responders exhibited persistence in remission from week 24 to week 52. Importantly, these patients did not stop their maintenance therapy, and indeed it was shown that drugs usually administered in Crohn’s disease do not affect MSC function [27].

Results of a clinical study on intravenous (iv) MSC administration as a therapeutic approach for chronic heart failure have been presented at the European Society of Cardiology Congress 2016 [28]. This phase IIa single-blind, placebo-controlled crossover clinical trial evaluated iv infusion of allogeneic ischemia-tolerant mesenchymal stem cells (itMSCs) in 22 patients with non-ischemic cardiomyopathy and a left ventricular ejection fraction of less than 40%. Patients were evaluated at baseline, at 90 days and at 180 days. No differences were found in the incidence of adverse events between the placebo and the itMSC-treated group. iv itMSC administration significantly improved several endpoints related to clinical efficacy, including the six-minute walk test (*p* = 0.02) and the Kansas City Cardiomyopathy Questionnaire (KCCQ) Clinical Summary score (*p* = 0.02). This study also suggests that intravenously administered itMSCs suppress inflammation, a critical pathogenic element in the progression of heart failure, as there was a statistically significant reduction in natural killer (NK) cells, which correlated with the improvement in left ventricular ejection fraction.

In another recent report [29], 18 patients with stable, chronic stroke were enrolled in a two-year, open-label, single-arm study to evaluate the safety and clinical outcomes of surgical intra-cranial transplantation of modified bone marrow-derived MSCs (SB623). Six patients experienced six serious adverse events associated with the transplantation procedure, but all recovered without sequelae. The sixteen patients who completed the 12 month-follow-up showed significant improvement for the European Stroke Scale, the National Institutes of Health Stroke Scale and the Fugl-Meyer motor function total score, while no changes were observed in the modified Rankin Scale. The area of magnetic resonance T2 fluid-attenuated inversion recovery signal change in the ipsilateral cortex 1 week after implantation significantly correlated with clinical improvement at 12 months. Thus, in this interim report, intracranial transplantation of MSCs was safe and associated with clinical improvement.

In summary, at present SSCs seem to be far ahead of PSCs in clinical applications. Clearly, the safety profile of PSCs still needs to be improved to allow their further development as therapeutic tools.

## 3. Allogenic vs. Autologous Cells

The use of allogeneic versus autologous cells represents an unsettled issue in SSC clinical applications. Studies in vitro and in vivo with MSCs showed similar efficacy of the two cell sources in suppressing immune response and stimulating tissue regeneration [30,31,32,33], and no serious adverse events were reported in clinical trials with allogeneic MSCs. Allogenic transplants generally need constant immunosuppression to prevent rejection of grafted cells. However, the above-cited clinical trials reporting successful results with MSCs for the treatment of pediatric GVHS, fistulae in Crohn’s disease and chronic heart failure were all using allogenic cells. MSCs have been considered immune privileged cells because express very low levels of MHC class I, no MHC class II and do not induce activation of allogeneic lymphocytes [34], However, allogenic MSCs are prone to cytotoxic lysis under inflammatory conditions in vitro, and induce the production of complement-activating antibodies in vivo [35]. Moreover, stimulated NK cells can lyse both autologous and allogeneic MSCs [36]. In vivo studies showed that MSC survival following transplantation is limited [37,38]. Therefore, persistent engraftment of transplanted cells does not seem to be a prerequisite for their therapeutic efficacy, suggesting that the observed clinical benefits result from a time-limited effect.

While the use of autologous MSCs may seem more desirable, none of the published trials in GVHD have employed autologous cells, because of the risk of transmitting tumor cells from the hematologic malignancy under treatment back to the recipient and of concerns about possible genetic abnormalities resulting from prior cytotoxic chemotherapy. Autologous MSCs have been used in other clinical applications, but in some pathologic conditions cells may harbor abnormalities associated with the disease being treated [39]. Obtaining a number of cells sufficient for clinical use may require 6–8 weeks of culture, a timeframe that may not be compatible with the clinical needs of individual patients, and autologous cells are not always suitable for efficient ex vivo expansion. On the other hand, allogenic cells are available “off the shelf,” also allowing better standardization and reducing costs of production. This would increase the number of patients that could benefit from such therapies. There is however, a need for reliable specific in vitro potency assays to predict the clinical efficacy of different cell preparations.

The development of broadly human leukocyte antigen (HLA)-compatible cell lines was theorized a decade ago [40]. Such cell lines could be generated either by ESCs [41] or by iPSCs [42] to meet most of the needs for HLA-I compatible cell and tissue transplants in the population. The feasibility and actual clinical applicability of such “super donors” needs further investigation.

## 4. Replacement vs. Regeneration

According to one early definition, Regenerative Medicine involves “the engineering and growth of functional biological substitutes in vitro and/or the stimulus to regeneration and remodeling of tissues in vivo for the purpose of repairing, replacing, maintaining or enhancing tissue and organ functions.” [43]. The early years of this novel discipline were dominated by the theory of “replacement”, and indeed, as reported above, this concept seems to work well with epithelial tissues such as the skin and the cornea. However, the results were less clear with more complex, slow-turnover tissues such as those in heart, the brain, or the liver. In animal studies, the disproportion between significant functional recovery and poor cell engraftment and persistence suggested an indirect primary mechanism other than the structural integration of transplanted cells into injured tissues. This was particularly evident in studies on experimental cell transplantation as a therapy for myocardial infarction [44]. The secretion of cytoprotective factors by MSCs was first reported by Gnecchi and colleagues [45]. In their experimental model, the finding that transplantation of genetically modified MSCs could prevent ventricular remodeling and reestablish heart function in less than 72 h following surgical myocardial infarction raised the possibility of an action other than a myogenic differentiation that would not take place in such a short time period. Indeed, similar improvements were shown in the same model, following injection of MSC-conditioned medium, pointing to paracrine signaling as the primary mechanism underlying the beneficial effect of cell therapy. It is now well established that stem cells transplanted into injured body sites secrete a variety of factors inhibiting apoptosis, enhancing angiogenesis, stimulating endogenous stem cells and inhibiting fibrosis, thus promoting tissue restitutio ad integrum [46,47]. The underlying molecular mechanisms are also being unraveled [48]. Regarding MSC-mediated immunomodulation, several soluble factors have been proposed to mediate the effect, including transforming growth factor-β1 (TGF-β1), prostaglandin E2 (PGE2), hepatocyte growth factor (HGF), indoleamine-pyrrole 2,3-dioxygenase (IDO), nitric oxide (NO) and interleukin-10 (IL-10). Although several secreted factors are involved in the immunomodulation of MSCs, their relative relationship remains unclear. The effect of soluble factors on the activity of MSCs may vary depending on the tissue of origin of the cells, on target cell phenotype and on the microenvironment. Indeed, the detailed mechanisms underlying the immune modulatory effects of MSCs are not yet sufficiently understood [49]. Though it is indisputable that MSC therapy contributes to immunosuppression, further elucidation of the detailed biological mechanisms involved in this process is required. It should also be noted that some cytokines or chemokines released from MSCs can act as promoters of inflammation. Understanding the delicate balance between beneficial and potentially harmful secreted factors is a key issue to successfully exploit the immune modulatory effects of MSCs [50].

## 5. Novel Technologies for Bioprocessing of Advanced Cellular Therapies

### 5.1. Automated vs. Non-Automated Procedures for Cell Culture

Once manipulated outside the body, cells constitute a drug—more specifically, an advanced therapy medicinal product (ATMP)—whose production is regulated by competent national and international authorities. Cell therapy products described in the previous chapters require various manufacturing methods [51]. The choice of the right cell culture system depends on various factors i.e., type of cells, scale of production, intended use, safety level of the laboratory, and the drug development strategy [52,53]. Proper selection of the production process strategy is the key factor in the commercial success of the drug product (Table 1).

In general, autologous cells require low-volume batch manufacturing processes, while allogenic cell production maximizes a single batch volume. Cell therapy products can be produced in automated, large volume and/or high-throughput bioreactor systems, but also by manual procedures performed by skilled personnel. The cell therapy product’s characteristics and its intended usage usually determine aspects of the production process. For example, the most rational choice for allogenic cell products targeting a high prevalence disease would be a large volume bioreactor system. On the other hand, bio-printed implants for tissue reconstruction would require an individual protocol, and customized non-automated production. Batch definition is a critical factor for planning the cell manufacturing process, and it is quite different for allogenic and autologous products. One batch of an autologous cell therapy product usually provides a single dose for one patient, while one batch of an allogenic product can provide hundreds or thousands of doses for multiple patients. Cell expansion of a personalized cell therapy product can be performed manually in a classical one-layer or multiple-layer two dimensions (2D) culture vessel, yielding up to hundreds of millions of cells. Some autologous products require a high-throughput production e.g., chimeric antigen receptor-T cells. In such cases, multiple bioreactors can work in parallel, providing a high number of low-volume batches of individual products. In the latter case, the system can be fully automatized in a similar way to large-volume bioreactor systems. Currently available large volume production systems are delivering the most cost-efficient products, due to the economy of scale, and significant reduction of space and personnel required for the production of a single dose. The key advantages of the automated systems are (i) reduction of operator-related errors and batch-to-batch variabilities; (ii) cross-contamination risk reduction in a low-grade biosafety environment; (iii) higher efficiency and traceability due to continuous monitoring and control of the cell culture parameters; (iv) time and personnel reduction; (v) the possibility of using a lower grade environment for the production process.

### 5.2. Defined vs. Non-Defined Culture Media

The quality of the reagents used for manufacturing of the cell therapy products has a critical influence on the purity and safety of the drug. For MSC production, standard protocols used for cell expansion require the use of bovine serum, usually not exceeding 15% of a total volume, since this ensures good adhesion of MSCs to most of the plastic surfaces and good cell growth. On the other hand, animal serum carries an infection risk. In addition, the bovine serum properties can vary between the batches, affecting both the reproducibility and quality of the product. Moreover, increasing demand could result in shortages of the product. In some cases, human platelet lysate can be used as a substitute for the bovine serum. However, platelet lysate has also an undefined composition, with large batch-to-batch variability and carries an infection risk. Defined media containing recombinant growth factors, selected proteins and amino acids, can improve the safety and reproducibility of ATMP production, but still suffer from drawbacks, such as impaired plastic adherence of MSCs [54]. Better understanding of the factors controlling cell adhesion will allow the development of more efficient production systems in the future.

## 6. From Cells to Cell Products: Introducing Extracellular Vesicles

As stated above, it is now fully appreciated that transplanted stem cells exert many of their pro-regenerative and immune modulatory effects via paracrine signaling. The traditional view of cell–cell communication consists in the release of bioactive molecules, resulting in a complex mixture whose local precise composition is largely undetermined. However, it has been known for many years that cells also secrete membrane vesicles, now named “extracellular vesicles (EVs)”, composed of a lipid bilayer including transmembrane proteins and enclosing cytoplasmic components [55,56,57,58]. EVs are best classified according to their intracellular origin, although the heterogeneity of these nanoparticles is very complex and only partially understood. Apoptotic bodies are released through outward blebbing and fragmentation of the cell membrane during the process of apoptosis, and exhibit a wide size range of 50–2000 nm. Shedding vesicles (also named microvesicles), ranging from 50 to 1000 nm in diameter, are released by direct outward budding of the plasma membrane through the disruption of cortical cytoskeleton. Exosomes are apparently less heterogeneous in volume, ranging in size from 30 to 120 nm, are derived from multivesicular bodies (MVBs), a late endosomal compartment, and are secreted via fusion of MVBs with the plasma membrane. Such tiny vesicles are complex biological machines that can convey signals by interacting at the cell surface, by internalization into endocytic compartments or by fusion with plasma membranes. EV complexity can be inferred by their composition, including proteins, nucleic acids and lipids, differing to variable extents depending on the tissue of origin, and on a variety of stimuli applied to the parent cells. More than 4000 different proteins and 2400 different RNAs are described in the exosome database ExoCarta, all of them potentially involved in the transmission of specific signals. In parallel with the recognition of their biological importance, EVs have become an object of increasing interest for possible clinical applications, and many authorities now believe they will replace their cells of origin as therapeutic tools, because of several reasons. Regarding safety, tumorigenic risk and ectopic colonization appear to be less of a concern with EVs than with living cells. Results in animal models suggest that local EV administration is feasible, e.g., for lung diseases (inhalant or intratracheal), to reach the central nervous system via the intranasal route, or as topical treatments of ocular diseases. Since EVs are not living cells reacting to different environments, but rather convey a definite set of signals, their effects should be both more predictable and reproducible. The regenerative and immunomodulatory effects of MSCs seem to be largely mediated by EVs (Table 2), and a case of successful treatment with MSC-EVs in a patient with steroid-resistant GVHD has been reported [59].

Administration of allogenic vs. autologous MSC-derived EVs should not be an issue, since these nanoparticles do not express MHC class I-II [72]. MSC-derived EVs can thus be produced by “waste” human tissue, such as Wharton jelly from the umbilical cord, and can be readily administered “off-the-shelf”. Specific cell targeting by EVs can increase the therapeutic index of EV-carried drugs, both by increasing drug concentration in the affected tissue and reducing unwanted distribution to other sensitive tissues and organs [73,74]. Clinical-grade EVs have been produced and tested in preliminary clinical trials on cancer immune therapy [75,76] (Table 3), and no safety concerns emerged both from past and ongoing EV-based clinical trials [77].

**Figure 1:**
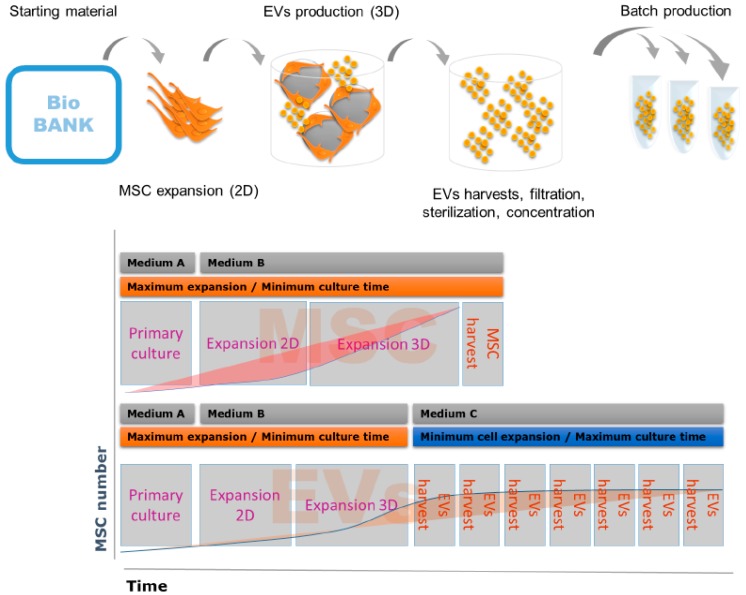
Bioproduction process of extracellular vesicles (EVs) from mesenchymal stem cells (MSCs).

Of note, EVs with therapeutic potential are being isolated both from ESC [81] and iPS-derived cell lines [82,83,84], suggesting that these nanoparticles might replace their cells of origin in some clinical applications, thus bypassing both ethical and safety concerns. EVs are also much easier to produce and cryopreserve under GMP requirements than living cells, with significant cost reductions, thus eliminating a major barrier to the diffusion of advanced therapies. Manufacturing of the MSC-EVs represents a good example, implying new standards in drug production and resulting in significant cost reductions without compromising product quality (Figure 1).

The entire production process, including final concentration and vial filling, can be performed in a fully automated and closed system. The quality control of the EVs bioproduction process starts from analysis of donor eligibility and includes all subsequent production steps (Table 4, Figure 1).

**Figure 2:**
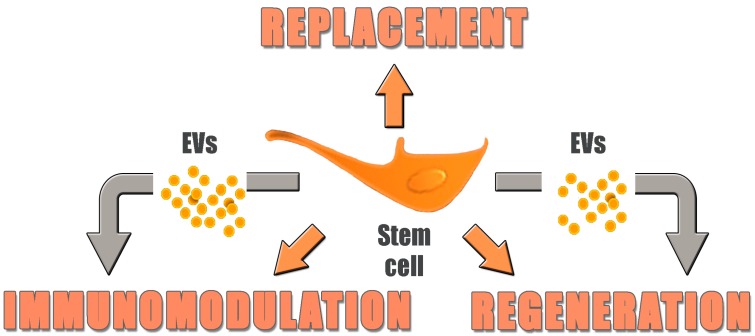
Stem cells vs. extracellular vesicles (EVs) as therapeutic tools. EVs seem to reproduce many of the regenerative and immunomodulatory effects of their cells of origin, but cells are still required to replace total loss of tissues.

The EVs (final product) are analyzed for size and quantity using state-of-the-art technology, such as nanoparticle tracking analysis and resistive pulse sensing analysis [85]. In addition, EV phenotyping must be performed to estimate EV homogeneity and quantity. Each EV batch is analyzed for several CD markers, including HLA class I/II (negative) and tetraspanin CD63, CD81, CD9 (positive). The above-mentioned tests should also be complemented by activity assays dedicated for the intended use of the EV drug e.g., in vitro co-culture with human immune cells to confirm the immunosuppressive effect of the EV batch. Unfortunately, such tests are difficult to standardize, and no reference assay has yet been established. A similar issue has been raised with MSC-based therapies [86,87]. EVs are thus opening a novel perspective in regenerative medicine, promising to speed up bench-to-bedside development with respect to living cells, as a result of their lower complexity, easier standardization and reduced production costs. However, it should be stressed that research on the use of EVs as therapeutic tool is still in its infancy, and that the biological properties of these nanoparticles are still poorly understood. For instance, little information is available on the variability of their complex cargo composition, and on the possibility of pre-determining such composition by the culture conditions of their cells of origin. The intracellular fate of EVs following their delivery into target cells is also largely unknown, but preliminary evidence suggests that it can vary depending on EV phenotype, resulting in profoundly different effects [88]. Most of all, their feasibility as therapeutic tools has been demonstrated only in animal models, while clinical experience is limited to the few above cited clinical trials in the field of adoptive immune therapy. Clearly, while EVs could eventually be used in place of their cells of origin in a series of pro-regenerative and immune modulatory therapeutic applications, whole cell transplantation will still be necessary to replace extensive or total loss of functional tissue, such as in the later stages of macular degeneration discussed above (Figure 2).

In conclusion, a series of successful clinical trials has propelled new confidence in the field of regenerative medicine, and new products are now being developed on firmer foundations, learning from past errors and taking advantage of recent technological advances. Presently, EVs are attracting much interest since they represent a potentially revolutionary therapeutic tool. However, much has still to be learned regarding their mechanisms of action, as well as regarding their efficacy and possible side effects in clinical practice.

## Figures and Tables

**Table 1:** Production process parameters characteristic for different advanced therapy medicinal products (ATMPs).

ATMP	Batch Volume	Primary Culture	Upstream	Downstream	Cost per Cell Batch
Tissue engineering	++	Manual	NA	NA	++++
Autologous MSCs	++	Manual	NA	NA	+++
Allogeneic MSCs	+++	Manual	Automated	Semi-automated	++
MSC-EVs	++++	Manual	Automated	Automated	+

MSCs: Mesenchymal stem cells; EVs: Extracellular vesicles (see text); Upstream process: Cell expansion and product harvesting from low volume starting material up to a large volume culture of the intermediate product; Downstream process: Intermediate product concentration, purification and packaging from large volume bioproduction system up to low volume final product vials. NA: not applicable; +: Very low; ++: Low; +++: Medium; ++++: High.

**Table 2:** Preclinical in vivo studies on the immune modulatory and regenerative effects of MSC-extracellular vesicles (EVs).

Source of EVs	Methodology	Results	Reference
**Rat BM-derived MSCs**	Rat Acute Kidney Injury (AKI) induced by gentamicin (G).	Improved renal histology and function.	[ 60]
**Human embryonic stem cell-derived MSCs**	Myocardial infarction in mice.	Reduced infarct area.	[ 61]
**Human BM-derived MSCs**	AKI induced by ischemia-reperfusion injury in rats.	Improved renal histology and function.	[ 62]
**Human BM-derived MSCs**	In vitro: EVs on cisplatin-induced apoptosis of human renal tubular epithelial cells.	In vitro: EVs up-regulated in cisplatin-treated human tubular epithelial cells anti-apoptotic genes, and down-regulated genes leading to cell apoptosis.	[63]
	In vivo: Cisplatin-induced AKI.	In vivo: Improved renal function and histology, improved survival.	
**Human UC-derived MSCs cultured under hypoxia**	Rat hindlimb ischemia model.	Improved blood flow recovery.	[ 64]
**Murine BM-derived MSCs**	Hypoxia-induced pulmonary hypertension in rats.	Inhibition of vascular remodeling and of hypoxic pulmonary hypertension.	[ 65]
**Human umbilical cord mesenchymal stem cells**	In vitro: treatment with cisplatin alone in NRK-52E cells.	In vitro: Reversal of cisplatin induced apoptosis and oxidative.	[66]
	In vivo: cisplatin-induced Acute Kidnay Injury (AKI) rat models. At 24 h after treatment with cisplatin, EVs injected into the kidneys.	In vivo: Improved kidney histology and biochemical parameters of kidney function.	
**Human BM-derived MSCs cultured under hypoxia**	Acute myocardial infarction rat model.	Improved blood flow recovery, reduced infarct size and preserved cardiac systolic and diastolic performance.	[ 67]
**Human UC-derived MSCs**	Rat model of skin deep second-degree burn wound.	EV-treated wounds exhibited accelerated re-epithelialization, with increased expression of CK19, PCNA, collagen I (compared to collagen III). Activation of Wnt/β-catenin by hucMSC-; Wnt4 was found in MSC-EVs, and promoted β-catenin nuclear translocation and activity to enhance proliferation and migration of skin cells.	[ 68]
**Human BM-derived MSCs**	DSS-induced colitis in mice.	Improved body weight and clinical score, reduced colon shortening, reduced TNFα, IL-1β and COX-2 expression in colon mucosa.	[ 69]
**Rat BM-derived MSCs**	TNBS-induced colitis in rats.	Improved body weight and clinical score, reduced colon shortening, improved histology, reduced TNFα, IL-1β, COX-2 and increased IL-10 in colon mucosa.	[ 70]
**Human BM-derived MSCs**	Hypoxic-ischemic injury of the preterm brain in lamb fetuses.	Improved brain function by reducing the total number and duration of seizures, and by preserving baroreceptor reflex sensitivity, tendency to prevent hypomyelination.	[ 71]

BM = Bone Marrow; UC = Umbilical Cord; DSS = Dextran sulfate sodium; TNBS = 2,4,6-trinitrobenzenesulfonic acid.

**Table 3:** Published clinical trials on EV-based therapies-modified from Ohno et al. [77].

Disease (No. of Patients)	EV Source	Outcome	Side Effects	Reference
Metastatic melanoma (15)	DCs autologous	MART1–HLA-A2 T-cell response and tumor shrinkage (1); minor response (1); mixed response (1); stabilization (2)	No major toxicity; minor inflammation, Grade 1 fever (5)	[78]
Non-small cell lung cancer (9)	DCs autologous	MAGE-specific T cell responses (3); NK cell lysis (2)	No major toxicity; moderate pain (1), swelling at injection site (8); mild fever (1)	[76]
Colorectal cancer (37)	Ascitic Fluid autologous	EVs + GM-CSF: cytotoxic T cell resp. to CAP-1 (76.9%); stabilization (1); minor response (1)	No major toxicity; moderate pain, swelling, pruritus at injection site (37); fever (1), fatigue (3) and nausea (1)	[79]
GVHD (1)	MSCs allogenic	GVHD symptoms improved; stabilization for several months. Patient died of pneumonia 7 months post exosome application	No major side-effects	[59]
Non-small cell lung cancer (41)	DCs autologous	N.D.	N.D.	[80]

DCs: dendritic cells; N.D.: No data; GM-CSF: granulocyte-macrophage colony-stimulating factor; GVHD: Graft-versus host disease; IFN-γ: interferon γ.

**Table 4:** Quality controls for bioprocessing of mesenchymal stem cell-derived extracellular vesicles.

QC Number	Tests
Starting material	
QC1	Documents check (including donor’s written consent) Donor’s medical history check Donor’s blood sample collection for microbiology and virus tests—results available within two weeks Transport time and transport environment monitoring Transport box and tissue container integrity check
QC2	Tissue visual inspection Fresh tissue viability and integrity test Transport medium microbiology test—results available within two weeks
Master bank	
QC3	Cell viability during primary growth continues monitoring of cell culture parameters: (pH, glucose, temp, humidity, CO_2_) Continues monitoring of cell viability and growth rate Continues monitoring of cell culture parameters: (pH, glucose, temp, humidity, CO_2_) Heterogeneity test: MSCs immunophenotyping (fluorescence-activated cell sorting (FACS))
QC4	Microbiology, endotoxins and mycoplasma tests—results available within two weeks
Working bank	
QC5	Cell recovery after thawing
QC6	Continues monitoring of cell viability and growth rate during EVs production Continues monitoring of cell culture parameters: (pH, glucose, temp, humidity, CO_2_) EV sizing and quantification (e.g., nanoparticle tracking analysis (NTA), tunable resistive pulse sensing (TRPS)) from every harvest, final pool and the final product EV activity test (e.g., T/B cell co-culture assay)
QC7	Final product test: microbiology and endotoxins—available within two weeks EV recovery and stability test after cryopreservation and storage at different temperature (sizing, phenotyping, activity)
